# Progressive Choreiform Movements in a Child: Early Recognition and Management of Sydenham Chorea

**DOI:** 10.5334/tohm.988

**Published:** 2025-02-06

**Authors:** Jason M. Jaronik, Nicholas A. Scott, Bruce D. Harley, Phillip L. Marsh, Hassaan A. Khan, Sufyan Zackariya, Anna M. Tincher, Anthony V. Thomas, Mahmoud D. Al-Fadhl, John R. Bales, Morgan C. Lain, Uzma Rizvi, Randall J. Bjork, Mark M. Walsh

**Affiliations:** 1 Department of Emergency Medicine, Saint Joseph Regional Medical Center, Mishawaka, Indiana, USA; 2 Department of Emergency Medicine, Memorial Hospital, South Bend, Indiana, USA; 3 Indiana University School of Medicine, South Bend, Indiana, USA; 4 Indiana University School of Medicine, Indianapolis, Indiana, USA; 5 Department of Mortality/Quality & Safety Data Review, Beacon Health System, South Bend, Indiana, USA; 6 Department of Neurology, Saint Joseph Regional Medical Center, Mishawaka, Indiana, USA

**Keywords:** Sydenham chorea, Rheumatic fever, Antistreptolysin, Chorea, Child

## Abstract

**Background:**

Sydenham chorea, a rare neurological manifestation of acute rheumatic fever, persists in developed countries due to rheumatogenic strains of group A streptococcus.

**Phenomenology shown:**

This case demonstrates the evolution from subtle early symptoms to definitive severe choreiform movements in Sydenham chorea in a 10-year-old female.

**Educational Value:**

This case highlights the importance of early recognition, multidisciplinary management, and vigilance in medication administration to optimize outcomes in rare conditions such as Sydenham chorea.

**Highlights:**

This case highlights the diagnostic and management challenges of Sydenham chorea, showcasing its progression from subtle early symptoms to definitive severe choreiform movements. It demonstrates the importance of early recognition, multidisciplinary care, and cautious medication administration to optimize outcomes in this rare neurological condition associated with rheumatic fever.

## Background

Sydenham chorea, though rare in developed countries due to widespread antibiotic access, remains a significant neurological manifestation associated with acute rheumatic fever [1,2]. This case highlights the challenges in diagnosing and managing this condition, emphasizing the importance of early identification and appropriate therapeutic strategies.

## Case Report

A 10-year-old girl presented to the ED with nonspecific, rapid involuntary movements of the extremities initially described as twitching. Two weeks earlier, she had streptococcal pharyngitis treated with amoxicillin. This history combined with her symptoms raised suspicion for Sydenham chorea. An antistreptolysin O titer (ASO) was ordered, and she was discharged with instructions to return if symptoms worsened. Over six days, her symptoms progressed to severe motor impairment, prompting her return. The ASO was elevated at 888 Todd units (normal: 200–310). Examination revealed sporadic choreiform movements of the head, neck, torso, and extremities with the left arm more affected compared to the right (see Video 1). She also demonstrated sporadic transitioning between normal speech and laughter.

**Video 1:** The video shows a 10-year-old female exhibiting sporadic choreiform movements of the arms, legs, torso, neck, and head consistent with Sydenham chorea.

Labs showed low creatinine (0.53 mg/dL), low ammonia (58 mcg/dL), and low valproic acid (40 mcg/mL). Her head CT and MRI were unremarkable. Echocardiography revealed aortic and mitral regurgitation. The combination of her clinical presentation and diagnostic workup met the Jones criteria for acute rheumatic fever. Treatment included 4.8 million units penicillin G benzathine, 60 mg prednisone daily, 500 mg valproic acid twice daily, and 1 mg haloperidol twice daily. Haloperidol was added to the treatment regimen primarily for its proven efficacy in reducing the involuntary movements associated with Sydenham chorea. After 18 days of admission, rehabilitation, and therapy, her symptoms had significantly improved, and she was discharged on a prednisone taper and 1 mg haloperidol twice daily. On follow-up evaluation, she reported complete resolution of symptoms and was able to attend school with no issues.

## Discussion

Although rare in developed nations, Sydenham chorea persists due to rheumatogenic group A streptococcal strains, requiring clinical suspicion after streptococcal infections [1]. Sydenham chorea can be difficult to identify because of its rarity and the non-specific nature of its early presentation. Also, it can develop up to several months after streptococcal pharyngitis [1,2]. Thorough history-taking of recent streptococcal pharyngitis and the presence of other features of rheumatic fever, such as carditis, arthritis, and erythema marginatum-type rash [3] will allow the clinician to pursue laboratory confirmation with an ASO.

This case demonstrated rapid progression from the non-specific initial presentation—which was likely mild, large-amplitude early choreiform movements—to the more severe definitive chorea. Elevated ASO confirmed the diagnosis, enabling early intervention. Collaboration with pediatric neurology allowed comprehensive management. The patient’s improvement highlighted the efficacy of our therapeutic approach.
